# Perioperative Suppression of Schwann Cell Dedifferentiation Reduces the Risk of Adenomyosis Resulting from Endometrial–Myometrial Interface Disruption in Mice

**DOI:** 10.3390/biomedicines10061218

**Published:** 2022-05-24

**Authors:** Xi Wang, Xishi Liu, Sun-Wei Guo

**Affiliations:** 1 Shanghai OB/GYN Hospital, Fudan University, Shanghai 200011, China; wangxi9508@163.com (X.W.); lxsdoc@hotmail.com (X.L.); 2 Shanghai Key Laboratory of Female Reproductive Endocrine-Related Diseases, Fudan University, Shanghai 200011, China

**Keywords:** adenomyosis, dedifferentiation, endometrial–myometrial interface disruption, JNK pathway, MEK/ERK pathway, pathogenesis, Schwann cell

## Abstract

We have recently demonstrated that endometrial–myometrial interface (EMI) disruption (EMID) can cause adenomyosis in mice, providing experimental evidence for the well-documented epidemiological finding that iatrogenic uterine procedures increase the risk of adenomyosis. To further elucidate its underlying mechanisms, we designed this study to test the hypothesis that Schwann cells (SCs) dedifferentiating after EMID facilitate the genesis of adenomyosis, but the suppression of SC dedifferentiation perioperatively reduces the risk. We treated mice perioperatively with either mitogen-activated protein kinase kinase (MEK)/extracellular-signal regulated protein kinase (ERK) or c-Jun N-terminal kinase (JNK) inhibitors or a vehicle 4 h before and 24 h, 48 h and 72 h after the EMID procedure. We found that EMID resulted in progressive SCs dedifferentiation, concomitant with an increased abundance of epithelial cells in the myometrium and a subsequent epithelial–mesenchymal transition (EMT). This EMID-induced change was abrogated significantly with perioperative administration of JNK or MEK/ERK inhibitors. Consistently, perioperative administration of a JNK or a MEK/ERK inhibitor reduced the incidence by nearly 33.5% and 14.3%, respectively, in conjunction with reduced myometrial infiltration of adenomyosis and alleviation of adenomyosis-associated hyperalgesia. Both treatments significantly decelerated the establishment of adenomyosis and progression of EMT, fibroblast-to-myofibroblast trans-differentiation and fibrogenesis in adenomyotic lesions. Thus, we provide the first piece of evidence strongly implicating the involvement of SCs in the pathogenesis of adenomyosis induced by EMID.

## 1. Introduction

Adenomyosis, defined as the presence of the endometrium within the myometrium, is a common gynecological disease and is one contributing factor to dysmenorrhea, abnormal uterine bleeding (AUB), heavy menstrual bleeding (HMB), and subfertility [1]. Despite its first description by the German pathologist Carl von Rokitans over 160 years ago [2], its pathogenesis and etiology are still poorly understood [3]. While invagination and metaplasia are the two most popular theories [4], unfortunately, neither of them has much support from either epidemiological or experimental data [5].

In light of extensive epidemiological reports that iatrogenic uterine procedures, such as induced abortion and dilatation and curettage (D&C), could increase the risk of developing adenomyosis [6,7,8,9,10], we proposed a new hypothesis, termed endometrial–myometrial interface (EMI) disruption (EMID) [11]. Subsequent animal experiments demonstrated that mechanically or thermally induced EMID can and does cause adenomyosis in mice [12]. More remarkably, the EMID hypothesis successfully predicted that the risk of developing adenomyosis depends on the mode and severity of EMID, and that the risk can be mitigated by perioperative intervention [12]. Importantly, the finding that EMID induces adenomyosis has also been independently confirmed very recently in another mouse model [13]. Thus, our mouse model also provides a perfect explanation of why iatrogenic uterine procedures are a risk factor for adenomyosis.

While the establishment of the mouse model of EMID-induced adenomyosis provides experimental proof of the EMID hypothesis, questions about its exact molecular mechanism and the optimal way to intervene would naturally arise. It is possible that the understanding of its underlying mechanisms may help to uncover the etiology and pathogenesis of adenomyosis due to other causes.

Of particular interest is the EMI, a term which has been used somewhat interchangeably with another term, junctional zone (JZ), although the latter was originally defined by Hricak in 1983 [14] as a low signal band between the endometrium and myometrium in T2-weighted magnetic resonance imaging (MRI). The EMI/JZ has been well documented to be an important uterine region in adenomyosis as well as in uterine function, and its change, viewed under MRI, has long been used as an aid for the diagnosis of adenomyosis [15]. Incidentally or not, Leyendecker’s tissue injury and repair (TIAR) hypothesis postulates that adenomyosis originates from uterine hyperperistalsis that causes tissue injury in the JZ [16].

It is generally thought that endometrial epithelial cells could invade into the disrupted myometrium through epithelial–mesenchymal transition (EMT) due to elevated local estrogen levels [17] within an EMI which experiences injury. While EMT has been shown to be involved in the progression of adenomyosis [18], so far, there has no direct proof of its involvement in the formation of adenomyotic lesions.

By all standards, EMI is more than just a demarcation that separates the endometrium from the myometrium, or a physical barrier that impedes the invasion of endometrial epithelial cells into the myometrium. As a start, it is the region where the endometrial stem cells reside [19,20]. It also serves as an origin of uterine contractions [21]. Unlike the endometrium or myometrium, EMI is densely innervated with peripheral nerves [22,23,24,25], maintained by ensheathing Schwann cells (SCs) that are derived from the neural crest [26]. SCs are the most important type of glial cells in the peripheral nerve system (PNS) and are distributed nearly ubiquitously in the human body [27]. Their abundance, coupled with their remarkable plasticity that is capable of dedifferentiating and re-differentiating following injury to the nerve, gives them important roles in tissue repair and in promoting cancer progression [26,28,29,30].

When the peripheral nerves are injured, SCs can dedifferentiate into immature SCs and acquire stemness [26,31,32]. In addition, SCs can also transdifferentiate into endoneurial fibroblasts [33], chromaffin cells [34], melanocytes [35], mesenchymal cells [36], and parasympathetic, sympathetic, enteric, GABAergic, glycinergic, serotoninergic and cholinergic neurons [37,38,39,40,41]. In view of their multifaceted roles after injury, we speculated that dedifferentiated SCs resulting from EMID might be coaxed and differentiated into endometrial epithelial cells through increased local production of estrogens due to platelet aggregation and hypoxia [42], and inflammatory cytokines and growth factors following tissue injury [43]. Furthermore, through EMT, these SC-turned endometrial epithelial and stromal cells (through EMT) may form the original focus of adenomyotic lesions. That is, SCs residing in the EMI, when injured, would become dedifferentiated and turn into endometrial epithelial and stromal cells, forming an original adenomyotic lesion in the disrupted myometrium.

Our in vitro experiments using a rat SC cell line indicated that SCs can indeed be coaxed into endometrial epithelial cells, which can be further differentiated into stromal cells through EMT (Wang et al., unpublished data). To test the hypothesis through an in vivo experiment, we designed this study, with the added goal of investigating whether perioperative suppression of SC dedifferentiation could reduce the risk of adenomyosis resulting from EMID in mice. It has been reported that Ras/Raf/extracellular-signal regulated protein kinase (ERK) and the c-Jun N-terminal kinase (JNK) are two important signaling pathways involved in the dedifferentiation of SCs [26,44], and inhibition of either pathway can prevent SCs from dedifferentiating [45,46]. Therefore, we tested our hypotheses in mice treated perioperatively with an MEK/ERK or JNK inhibitor.

## 2. Materials and Methods

### 2.1. Animals and Chemicals

One hundred and fifty-three female BALB/c mice, 6–8 weeks old, were purchased from Shanghai Jie Sijie Laboratory Animal Company (Shanghai, China) and used for this study. All mice were maintained under controlled conditions with a light/dark cycle of 12/12 h and had access to food and water ad libitum. All procedures were performed under the guidelines of the National Research Council’s Guide for the Care and Use of Laboratory Animals [47] and approved by the Institutional Experimental Animals Review Board of Shanghai OB/GYN Hospital (No. 2018-035, approved on 28 March 2018), Fudan University.

The MEK/ERK inhibitor U0126 and the JNK inhibitor SP600125 were purchased from Sigma (St. Louis, MO, USA). Both U0126 and SP600125 were dissolved in a DMSO/PEG300/Tween-80/saline mixture at a 1:4:0.5:4.5 ratio (by volume) at a dose volume of 10 μL/g bodyweight per day. We chose this dosage on the basis of previous reports [48,49,50,51].

### 2.2. Experimental Procedures

#### 2.2.1. Experiment 1

**Figure 1:**
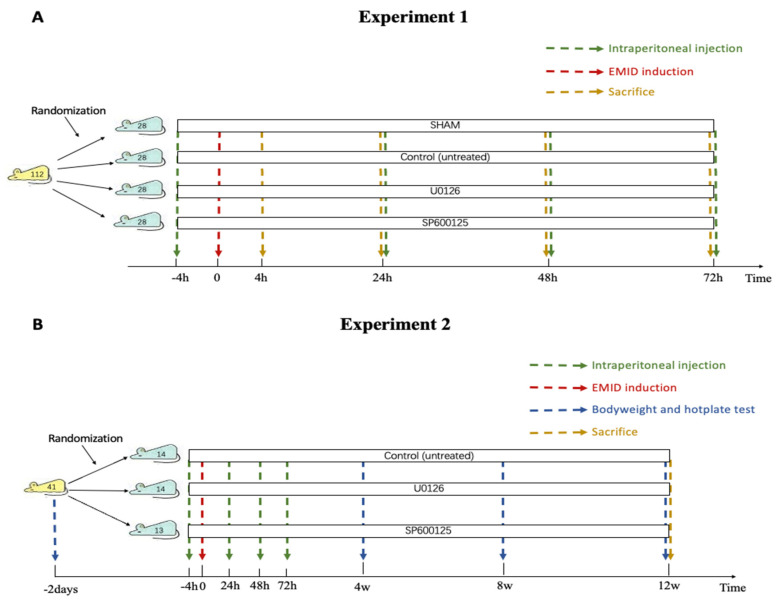
Schematic illustration of the experimental designs: Experiment 1 (**A**) and Experiment 2 (**B**).

To investigate whether SCs in the EMI undergo dedifferentiation after EMID induction and whether the two inhibitors can prevent SCs in the EMI from dedifferentiating, 112 sexually mature female BALB/c mice were randomly divided into 4 groups: sham (*n* = 28), CTL (control, vehicle only; *n* = 28), U0126 (MEK/ERK inhibitor, *n* = 28) and SP600125 (JNK inhibitor, *n* = 28). After 2 weeks of acclimatization, EMID was induced mechanically on one of two uterine horns, chosen at random, of each mouse for all mice from the CTL, SP600125 and U0126 groups as described previously [12]. The mice from the sham group received an incision identical to that of the other 3 groups but without the EMID procedure. Mice from different groups were intraperitoneally injected with different drugs 4 h before and 24 h, 48 h and 72 h after the induction procedure. Mice in the U0126 and SP600125 groups received daily treatment with U0126 (5 mg/kg bodyweight) and SP600125 (20 mg/kg bodyweight), respectively, while both the sham and CTL groups received a daily administration of a vehicle buffer of the same volume in an identical manner. Seven mice from each group were then sacrificed at 4 h, 24 h (before the second injection), 48 h and 72 h after the EMID or sham procedure. The uteri were collected and fixed in 4% neutral-buffered formalin for histological, immunohistochemical and immunofluorescence examinations. The design of this experiment is schematically illustrated in Figure 1A.

#### 2.2.2. Experiment 2

Forty-one sexually mature female BALB/c mice were randomly divided into 3 groups: CTL (control, *n* = 14), U0126 (*n* = 14) and SP600125 (*n* = 13). After 2 weeks of acclimatization, the bodyweight was recorded for all mice, and a baseline hotplate test was administered before the EMID induction procedure.

The EMID was performed mechanically on one of the two uterine horns, randomly chosen, of each mouse as described previously [12]. Mice from different groups were intraperitoneally injected with different compounds 4 h before and 24 h, 48 h and 72 h after the EMID procedure. Mice in the U0126 and SP600125 groups received a daily treatment of U0126 (the same as in Experiment 1) and SP600125 (the same as in Experiment 1), respectively, while the CTL group received a daily injection of the vehicle buffer at the same volume in the same manner. To assess the extent of adenomyosis-associated hyperalgesia, a hotplate test was performed with a commercially available Hot Plate Analgesia Meter (Model BME-480, Institute of Biomedical Engineering, Chinese Academy of Medical Sciences, Tianjin, China) as reported previously [12], on all mice before and every 4 weeks after the EMID procedure until sacrifice. Three months after the EMID procedure, all mice were sacrificed by cervical vertebra dislocation, and all their uterine horns were harvested and processed for further histological and immunohistochemistry analysis. The experimental design is shown in Figure 1B.

### 2.3. Hematoxylin–Eosin (H&E), Immunohistochemistry Staining (IHC) and Immunofluorescence

All uterine horns were fixed in 4% neutral-buffered formalin and then embedded vertically in paraffin. Each uterine horn was divided into 5–6 roughly equal-sized segments depending on the size of the uterine horns, and each tissue block was sliced into serial 4-μm sections. The first slides were used for H&E staining to evaluate the presence or absence of adenomyotic lesions or where the EMID procedure was performed. Five to six slides were evaluated for each mouse for confirmation. The depth of infiltration of the ectopic endometrium into the myometrium was evaluated according to the criteria of Bird et al. [52], as previously described [12]. Briefly, the infiltration of the ectopic endometrium tissues into the myometrium was classified into three grades (1, 2 and 3) depending on the depth of infiltration, involving the superficial, mid- or beyond mid-myometrium, respectively. For analytic purposes, Grade 0 was assigned when no ectopic endometrium was observed in the myometrium, i.e., no adenomyosis was present.

For the IHC and immunofluorescence analyses, slides were deparaffinized in xylene and rehydrated in a graded alcohol series. The slides were then heated to 98 °C in a citric acid buffer (pH 6.0) or EDTA (pH 9.0) for antigen retrieval. All sections were cooled naturally at room temperature and then incubated with 5% goat serum for 1 h at room temperature. Slides were then incubated overnight at 4 °C with primary antibodies (Table 1). On the next day, slides for IHC were incubated with the secondary antibody (JieHao Biotechnology, Shanghai, China) for 30 min at room temperature. The bound antibody complexes were stained for 3–5 min until they were appropriate for microscopic examination with diaminobenzidine (DAB) (JieHao Biotechnology, Shanghai, China) and then counterstained with hematoxylin. Five randomly selected images of each slide were obtained by an Olympus microscope (Olympus BX51, Olympus, Tokyo, Japan) at 400× magnification. Cells stained with tan-yellow colors in conjunction with the presence of violet-blue-colored nuclei were considered to be positive, and the mean density of staining intensity was acquired using Image Pro-Plus 6.0 (version 6.0.0.206; Media Cybernetics, Bethesda, MD, USA). Slides for immunofluorescence were incubated with donkey anti-mouse or donkey anti-rabbit IgG antibodies for 1 h at 37 °C and then stained with DAPI. Images were obtained by a laser scanning confocal microscope (Leica TCS SP5 Confocal Microscope, Leica, Solms, Germany). Cells with red-colored staining coupled with the presence of blue-colored nuclei were considered to be E-cadherin-positive, while cells with yellow-colored staining accompanied by the presence of blue nuclei were considered to be E-cadherin/vimentin dual-positive. The number of E-cadherin+ cells or E-cadherin/vimentin dual-positive cells within the myometrium was quantitated at 400× magnification, as they were present in the myometrium. For each mouse, 4 slides per marker were used for IHC analysis, and for the immunofluorescence analyses, 2–3 slides were evaluated for each mouse, and their averages were used.

To minimize any bias, the group identity of the slide that the scorer (X.W.) was evaluating was deliberately concealed from the scorer (X.W.); hence, she was practically blinded.

### 2.4. Masson Trichrome Staining

Three to six slides from each mouse were used to evaluate the extent of fibrosis in the lesions by Masson trichrome staining, as described previously [12]. Routine deparaffinization and rehydration procedures were performed as described above. The slides were immersed in Bouin’s solution at 37 °C for 2 h and were stained using the Masson’s Trichrome Staining kit (Baso, Wuhan, China) following the manufacturer’s instructions. Five randomly selected images of each sample were obtained by an Olympus microscope (Olympus, Tokyo, Japan) at 400× magnification. The proportion of collagen fibers, stained in blue, was calculated by Image Pro-Plus 6.0 (version 6.0.0.206; Media Cybernetics, Bethesda, MD, USA). Again, to minimize any bias, the evaluator was unaware of the group identity of the mouse she was evaluating and was practically blinded.

### 2.5. Statistical Analysis

Wilcoxon’s and Kruskal’s tests were used to make a comparison of the distributions of the continuous variables between or among two or more groups. Pearson’s correlation coefficient was calculated to evaluate the correlation, if any, between two IHC measurements. Multiple linear regression analyses incorporating the time of tissue harvest (4 h, 24 h, 48 h and 72 h), EMID induction (yes or no), treatment group (SP600125, U0126 or none) and their interactions were conducted to identify which factors were associated with the IHC staining levels. We noted that the use of linear regression used the data more efficiently and minimized the need for multiple testing. Here, *p*-values of less than 0.05 were considered to be statistically significant. All computations were made with Prism 9 software.

## 3. Results

### 3.1. Perioperative Administration of U0126 or SP600125 Prevents SCs in the EMI from Dedifferentiation after EMID Induction

**Figure 2:**
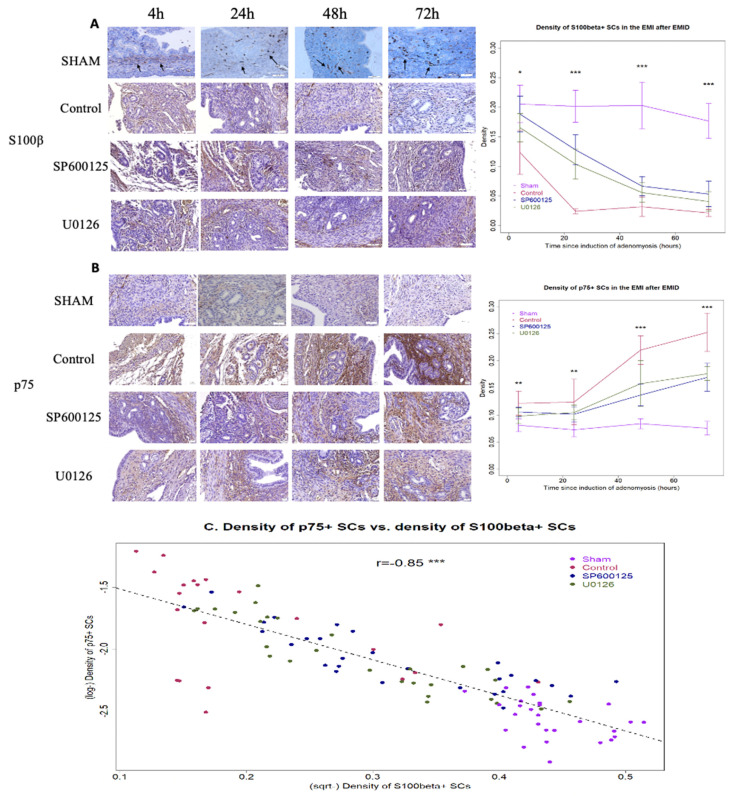
Perioperative administration of either SP600125 or U0126 abrogates EMID-induced SC dedifferentiation in the EMI region. Representative immunostaining results for S100β staining (**A**) and p75 staining (**B**) in the EMI from the sham, CTL, SP600125 and U0126 groups of mice, along with multivariate regression analysis summarizing the staining level at different times (right-hand panel). (**A**,**B**). The black arrows point to S100β+ SCs in the EMI (**A**). Magnification = 400×; scale bar = 50 μm. (**C**) The density of S100β+ SCs in the EMI correlated negatively with that of p75+ SCs at all time points (r = −0.85, *p* < 2.2 × 10^−16^). The solid line represents the regression line. Symbols for statistical significance levels: *: *p* < 0.05; **: *p* < 0.01; ***: *p* < 0.001, all according to Kruskal’s test.

All mice survived Experiment 1. IHC analysis was performed for S100β and p75, which are markers of mature SCs and dedifferentiated SCs [53,54,55,56,57], respectively. In mice from the sham group, we found that S100β-positive SCs were abundant in the EMI (Figure 2A), concomitant with fewer and more sporadic p75-positive SCs in the EMI and, to a lesser extent, in the stromal component (Figure 2B). The density of S100β+ SCs and of p75+ SCs was more or less constant 4 to 72 h after the EMID procedure (Figure 2A,B).

In contrast, the density of S100β+ SCs was progressively reduced (Figure 2A) and that of p75+ SCs was progressively elevated (Figure 2B) in the EMI from 4 to 72 h after the EMID induction, suggesting that EMID in itself resulted in SC dedifferentiation in the EMI. At all time points, there was a significant difference in the density of both S100β+ SCs and of p75+ SCs among the four groups of mice (all *p*-values ≤ 0.010; Figure 2A,B). Multivariate regression analysis incorporating the time of evaluation, whether or not the mice received the induction procedure, and whether or not they received SP600125 or U0126 treatment indicated that the EMID procedure resulted in a significant and progressive reduction in the density of S100β+ SCs (*p* = 0.023, *R*^2^ = 0.84; Figure 2A) but a significant and progressive increase in the density of p75+ SCs (*p* = 2.3 × 10^−13^, *R*^2^ = 0.82; Figure 2B) irrespective of the SP600125 or U0126 treatment. For all groups of mice and at all time points, the density of S100β+ SCs in the EMI correlated negatively with that of p75+ SCs (r = −0.85, *p* < 2.2 × 10^−16^; Figure 2C), indicating that the increase in dedifferentiated SCs was concomitant with a reduction in SCs losing their myelination marker.

Within mice with EMID-induced adenomyosis, the density of S100β+ SCs reduced progressively with time (*p* = 1.9 × 10^−5^), but treatment with SP600125 or U0126 resulted in a significant increase in the density of S100β+ SCs in the EMI (*p* = 2.8 × 10^−8^ and *p* = 1.9 × 10^−5^, respectively, *R*^2^ = 0.76; Figure 2A). Consistently, the density of p75+ SCs increased progressively with time (*p* < 2.2 × 10^−16^), but treatment with SP600125 or U0126 resulted in a significant decrease with time in the density of p75+ SCs in the EMI (*p* = 0.0002 and *p* = 0.0035, respectively, *R*^2^ = 0.75; Figure 2B).

Taken together, these data indicate that perioperative administration of either SP600125 or U0126 abrogates EMID-induced SC dedifferentiation in the EMI region of mice with adenomyosis.

### 3.2. Perioperative Administration of U0126 or SP600125 Reduces the Presence of Epithelial Cells in the Myometrium and Reverses Supsequent EMT after EMID Induction

**Figure 3:**
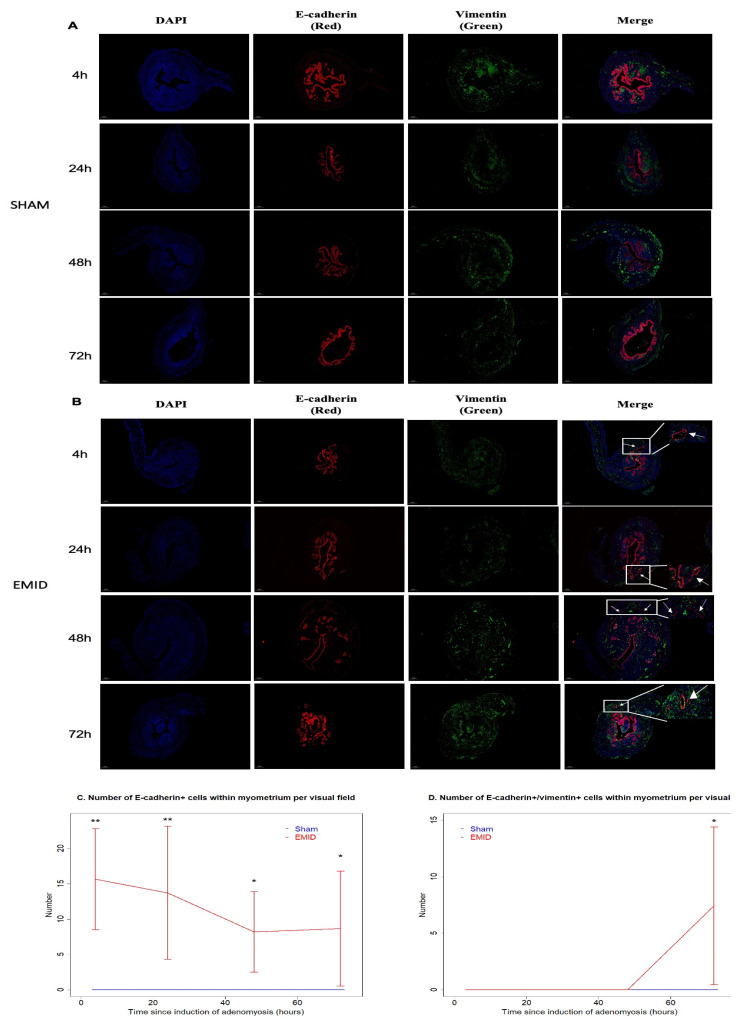
Representative photomicrographs of immunofluorescence staining suggestive of the occurrence of EMT in the myometrium after EMID. Tissue samples of uterine horns from mice in the sham group (**A**) and from mice that underwent EMID induction (**B**) were double-stained with antibodies against E-cadherin (red) and vimentin (green). DAPI was used as a nuclear stain (blue). Yellow-colored staining in the merged panel indicates the co-expression of both E-cadherin and vimentin. The arrows point to the E-cadherin-positive or E-cadherin and vimentin dual-positive cells in the myometrium. Enlarged versions of the images in the rectangle are shown in the upper/lower right corner in the merged panel (**B**). All images were captured using a confocal microscope at a magnification of 50 ×. Scale bar = 200 μm (except for the enlarged versions, which are shown in the upper/lower right corner in the merged panel). Dynamic changes in the number of E-cadherin+ cells (**C**) and E-cadherin and vimentin dual-positive cells (**D**) per visual field in the myometrium after induction at a magnification of 400×. Scale bar = 25 μm. Symbols for statistical significance levels: *: *p* < 0.05; **: *p* < 0.01, all according to Wilcoxon’s test.

To see whether EMID would induce EMT with the subsequent presence of epithelial-like cells in the myometrium, we also performed a dual immunofluorescence staining using antibodies against E-cadherin (an epithelial cell marker) and vimentin (a stromal cell marker) in the uteri of mice in the sham (no EMID, laparotomy only) and CTL (received EMID but otherwise untreated) groups. We found that E-cadherin+ and E-cadherin and vimentin dual-positive cells in the myometrium were completely absent in the sham group at 4 h, 24 h, 48 h and 72 h after EMID induction (Figure 3A). In contrast, E-cadherin-positive epithelial cells, which are normally confined to the endometrial epithelium and stroma, were found in the myometrium in the CTL mice with or without glandular morphology at 4 h, 24 h, 48 h and 72 h after EMID induction (Figure 3A). Additionally, E-cadherin and vimentin dual-positive cells with glandular morphology were also found in the myometrium at 72 h after, but not before, EMID (Figure 3B), indicating that EMT indeed occurred in these epithelial cells in the myometrium. The average number of E-cadherin+ cells in the myometrium in the CTL group at 4 h, 24 h, 48 h and 72 h after EMID induction were 15.6 (±7.1), 13.7 (±9.4), 8.2 (±5.7) and 8.6 (±8.1) per high-power field, respectively, which were significantly more than those from the sham group (*p* = 0.001, *p* = 0.004, *p* = 0.011 and *p* = 0.03, respectively; Figure 3C). Moreover, at 72 h after EMID induction, there was a sudden surge in E-cadherin and vimentin dual-positive cells in the myometrium in mice in the CTL group, significantly more than that in the sham group (7.4 ± 7.0 vs. 0.0 ± 0.0, *p* = 0.030; Figure 3D).

**Figure 4:**
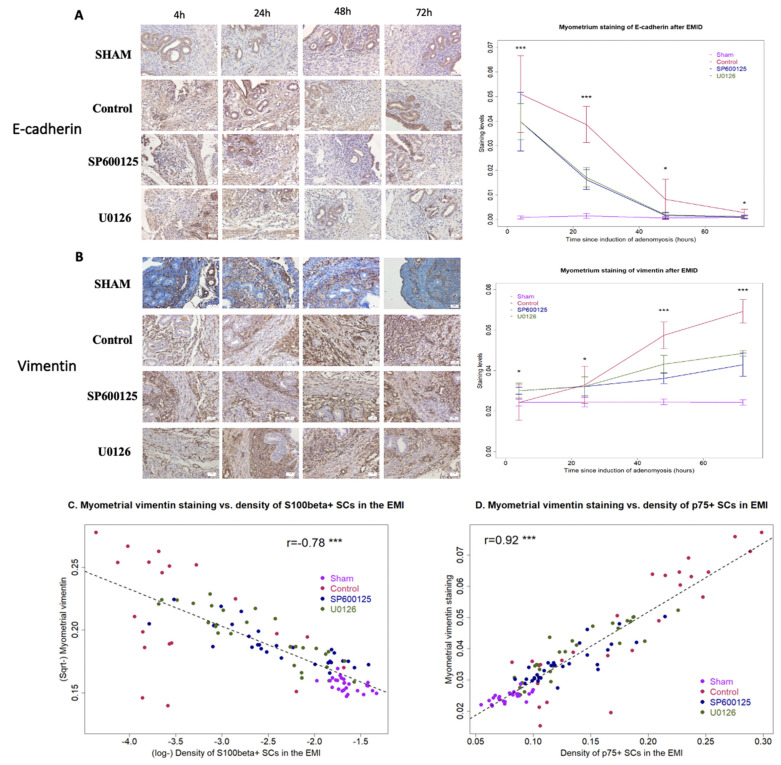
Perioperative administration of either SP600125 or U0126 reverses EMID-induced upregulation of epithelial cells and EMT in the myometrium. Representative immunostaining results for E-cadherin staining (**A**) and vimentin staining (**B**) in the myometrium from the sham, CTL, SP600125 and U0126 groups of mice, along with multivariate regression summarizing the staining level at different times (right-hand panel). (**A**,**B**). Magnification = 400×; scale bar = 50 μm. Scatter plot showing the density of S100β+ SCs in the EMI vs. myometrial vimentin staining (**C**) The density of p75+ SCs in the EMI region vs. myometrial vimentin staining (**D**). (**C**,**D**). The dotted line represents the regression line. Pearson’s correlation coefficient is shown, along with its statistical significance level. Symbols for statistical significance levels: *: *p* < 0.05; ***: *p* < 0.001. In (**A**,**B**), Kruskal’s test was used.

To see whether EMID-induced SC dedifferentiation yielded any presence of epithelial cells in the EMI region, we next performed IHC analysis of E-cadherin and vimentin in the myometrium regions harvested at different time points after the EMID. At all time points, there was a significant difference in the myometrial staining of both E-cadherin and vimentin among the four groups of mice (all *p*-values ≤ 0.033; Figure 4A,B). While in mice in the sham group (without EMID), the E-cadherin staining was essentially negative in the myometrium at all time points (all *p*-values ≥ 0.38 at 24 h, 48 h and 72 h as compared with 4 h; Figure 4A), the EMID procedure resulted in significantly elevated E-cadherin staining in the myometrium according to multivariate linear regression (*p* < 2.2 × 10^−16^, *R^2^* = 0.84; Figure 4A). Similarly, the vimentin staining was weak in the myometrium from mice in the sham group at all time points (all *p*-values ≥ 0.73 at 24 h, 48 h and 72 h as compared with 4 h; Figure 4B). In contrast, the EMID procedure resulted in significantly elevated vimentin staining in the myometrium in a time-dependent manner (*p* < 2.2 × 10^−16^, *R*^2^ = 0.86; Figure 4B). The elevated staining of vimentin induced by EMID was consistent with the immunofluorescence staining result and its time-dependency, as there was elevated vimentin staining in the myometrium at 48 h and 72 h as compared with 4 h and 24 h after EMID induction (Figure 3), strongly suggestive of EMT. The density of S100β+ SCs in the EMI region was found to be negatively correlated with the myometrial vimentin staining (*r* = −0.78, *p* < 2.2 × 10^−16^; Figure 4C), while the density of p75+ SCs in the EMI region was found to be positively correlated with the myometrial vimentin staining (*r* = 0.92, *p* < 2.2 × 10^−16^; Figure 4D).

Within mice that received EMID, the vimentin staining in the myometrium increased time-dependently (*p* < 2.2 × 10^−16^), but perioperative treatment with SP600125 or U0126 resulted in significantly reduced vimentin staining in a time-dependent fashion (*p* = 7.1 × 10^−7^ and *p* = 4.8 × 10^−10^, *R*^2^ = 0.81; Figure 4B).

Taken together, these data suggested the increased presence of epithelial-like cells in the myometrium after EMID with progressive EMT. Perioperative treatment with either SP600125 or U0126 abrogated the presence of epithelial-like cells in the myometrium and arrested subsequent EMT.

### 3.3. Perioperative Administration of U0126 or SP600125 Reduces the Risk of EMID-Induced Adenomyosis

In light of the above evidence for the involvement of SCs in EMID-induced adenomyosis, we next performed a mouse experiment to see whether inhibition of the ERK or JNK signaling pathway in the perioperative window can reduce the risk of developing adenomyosis.

All but one mouse in the CTL group survived the experiment. The CTL mouse died prior to the EMID procedure, probably due to bullying by the other mice in the same cage. During the entire course of the experiment, all three groups of mice gained weight during the course of the experiment, and no statistically significant difference in bodyweight was found among the three groups of mice measured at all four time points (all *p*-values ≥ 0.24).

**Figure 5:**
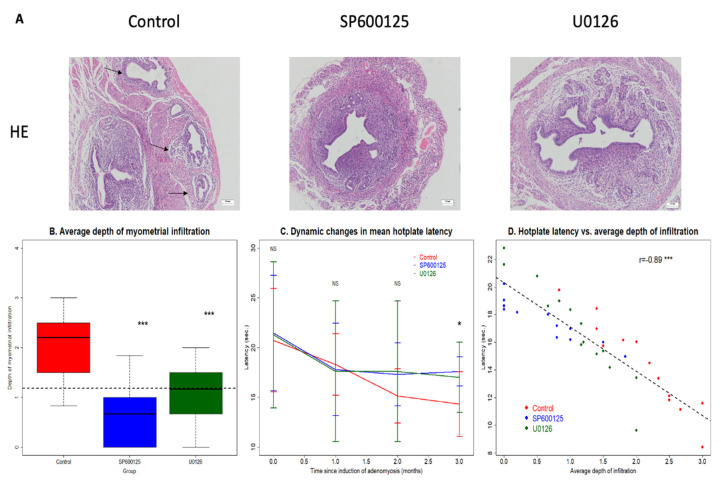
Perioperative administration of SP600125 and U0126 reduces the risk of endometrial–myometrial interface disruption (EMID)-induced adenomyosis. (**A**) Representative images of uterine tissues from the uterine horn subjected to EMID in mice from the three groups. The images in the SP600125 and U0126 groups are from mice without adenomyosis. Magnification = 100×; scale bar = 200 μm. The black arrows point to adenomyotic lesions. (**B**) Boxplot of the average grade of myometrial infiltration in uterine tissues from mice in the three groups. (**C**) Average hotplate latency, tested at the indicated times, in the three groups. (**D**) The hotplate latency correlated negatively with the average depth of myometrial infiltration (r = −0.89, *p* = 1.9 × 10^−14^). The solid line represents the regression line. Symbols for statistical significance levels: NS: *p* > 0.05; *: *p* < 0.05; ***: *p* < 0.001. In (**B**), Wilcoxon’s test was used (against the control). In (**C**), Kruskal’s test was used.

Three months after the EMID procedure, we found adenomyotic lesions in all CTL mice (13/13 = 100%), but eight in the SP600125 group (8/13 = 66.5%) and 12 in the U0126 group (85.7%). Thus, the incidence of adenomyosis in the SP600125 group, but not the U0126 group (*p* = 0.48), was significantly lower than that in the CTL group (*p* = 0.039; Figure 5A, Table 2). However, the average grade of myometrial infiltration of adenomyotic lesions in both the SP600125 and U0126 groups was significantly lower than that in the CTL group (*p* = 0.0002 and *p* = 0.001, respectively; Figure 5B and Table 2). The maximum depth of infiltration of both treatment groups, which correlated positively with the mean depth (*r* = 0.82, *p* = 7.3 × 10^−11^), was also significantly lower than that of the CTL group (*p* = 0.007 and *p* = 0.028, respectively). That is, even though the incidence of adenomyosis between the U0126 and the CTL groups was not statistically significantly different, the former group had significantly shallower infiltration than the latter group.

The hotplate latency in all groups decreased, but only significantly in the CTL mice (*p* = 0.006), and not in the SP600125 and U0126 mice (*p* = 0.15 and *p* = 0.091, respectively; Figure 5C). This is due to (1) the greater magnitude of reduction in the CTL group (30.9% as compared with that prior to the induction) than in either the SP600125 (18.0%) and U0126 (200%) groups and (2) the stabilization of the reduction in latency 1 month after the induction in the latter two groups. Multiple linear regression incorporating baseline latency, bodyweight at sacrifice and dummy variables representing the group identities indicated that at 3 months after induction, mice in the SP600125 and U0126 groups both had significantly longer latency than that of the CTL mice (*p* = 0.0057 and *p* = 0.023, respectively; *R*^2^ = 0.21; Figure 5C).

Consistently, the hotplate latency at the end of the experiment correlated negatively with the average depth of myometrial infiltration (*r* = −0.89, *p* = 1.9 × 10^−14^; Figure 5D) and with the maximum infiltration depth (*r* = −0.70, *p* = 4.9 × 10^−7^).

### 3.4. Perioperative Administration of U0126 and SP600125 Arrests the Progression of Adenomyosis 

**Figure 6:**
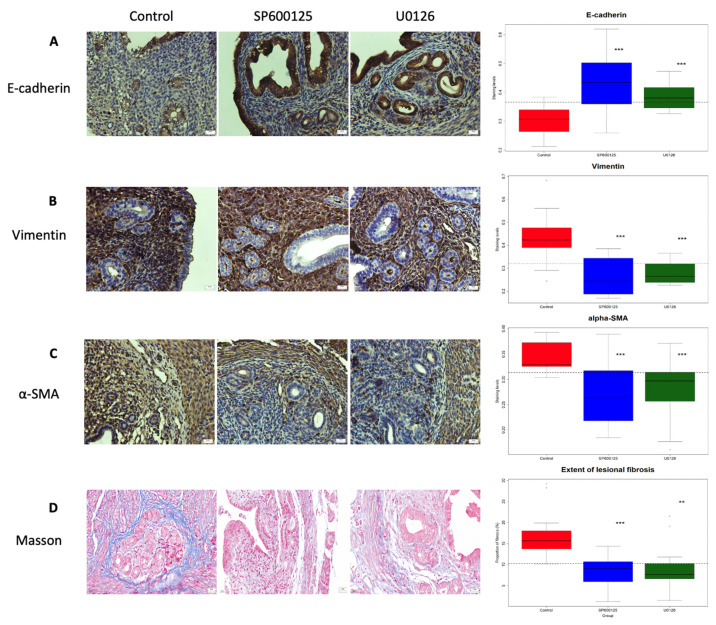
Perioperative administration of U0126 or SP600125 reduces the extent of lesion fibrosis and disease progression. Representative immunostaining results for E-cadherin staining (**A**), vimentin staining (**B**), α-SMA staining (**C**) and Masson staining (**D**) in the uteri from the three groups of mice, along with boxplots summarizing the staining data (right-hand panel). (**A**–**D**) Magnification = 400×; scale bar = 50 μm. Symbols for statistical significance levels: **: *p* < 0.01; ***: *p* < 0.001, all according to Wilcoxon’s test (against the control).

We next evaluated the staining of key markers in EMT and fibroblast-to-myofibroblast trans-differentiation (FMT), as well as the extent of fibrosis in adenomyotic lesions. We found that, compared with the CTL group, perioperative intervention with SP00125 and U0126 resulted in significantly increased E-cadherin staining (*p* = 0.00099 and *p* = 0.0002, respectively; Figure 6A) but significantly decreased vimentin staining in the epithelial component (*p* = 0.0002 and *p* = 0.0013, respectively; Figure 6B). In addition, the α-SMA staining in the adenomyotic stromal component was significantly reduced in both intervention groups (*p* = 0.0002 and *p* = 0.0003, respectively; Figure 6C).

Consistently, we found that the fibrotic content in the adenomyotic lesions was significantly reduced in both the SP600125 and U0126 groups as compared with CTL group (*p* = 1.3 × 10^−5^ and *p* = 0.0016, respectively; Figure 6D).

**Figure 7:**
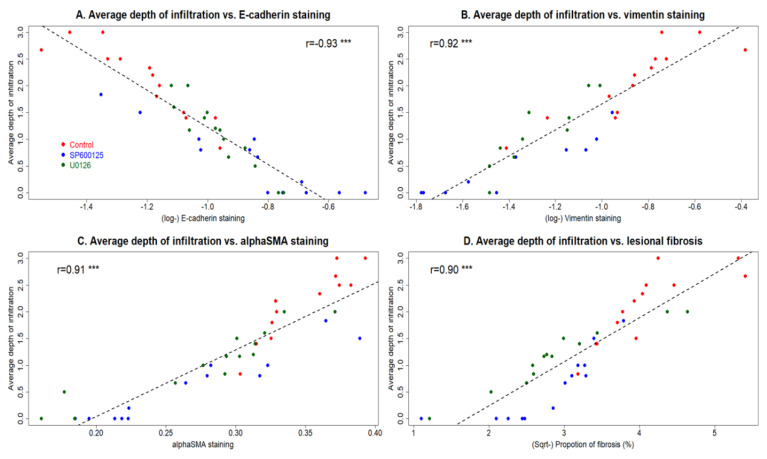
The perioperative intervention with SP600125 and U0126 retarded the development of adenomyosis through the suppression of EMT, FMT and fibrogenesis. The average depth of myometrial infiltration correlated negatively with the E-cadherin staining levels (*r* = −0.93, *p* < 2.2 × 10^−16^) (**A**) but positively with the vimentin staining levels (*r* = 0.92, *p* = 2.6 × 10^−14^) (**B**) in the adenomyotic epithelial component. The average depth of myometrial infiltration correlated positively with the α-SMA staining levels (*r* = −0.91, *p* = 6.5 × 10^−14^) (**C**) and the extent of lesional fibrosis (*r* = 0.90, *p* = 1.1 × 10^−14^) (**D**). The solid line represents the regression line. Symbol for statistical significance level: ***: *p* < 0.001.

Remarkably, the average depth of myometrial infiltration correlated negatively with the E-cadherin staining levels but positively with the vimentin staining levels in the adenomyotic epithelial component (*r* = −0.93, *p* < 2.2 × 10^−16^, and *r* = 0.92, *p* = 2.6 × 10^−14^; Figure 7A,B). In addition, it correlated highly and positively with the α-SMA staining levels as well as the extent of lesional fibrosis (*r* = −0.91, *p* = 6.5 × 10^−14^, and *r* = 0.90, *p* = 1.1 × 10^−14^; Figure 7C,D).

Taken together, these data suggest that the perioperative intervention with SP600125 and U0126 retarded the development, or establishment in some cases, of adenomyosis through hindering EMT, FMT and fibrogenesis.

## 4. Discussion

In this study, we found that EMID resulted in progressive SC dedifferentiation, concomitant with an increased abundance of epithelial cells in the myometrium and subsequent EMT. This EMID-induced change was abrogated significantly through perioperative administration of JNK or MEK/ERK inhibitors. Consistently, perioperative administration of the JNK inhibitor SP600125 reduced the incidence by one-third, in conjunction with reduced myometrial infiltration of the adenomyosis induced by EMID and alleviation of adenomyosis-associated hyperalgesia. While perioperative treatment with the MEK/ERK inhibitor U0126 did not reduce the incidence of adenomyosis significantly, both treatments significantly decelerated the establishment of adenomyosis and the progression of EMT, FMT and fibrogenesis in adenomyotic lesions. To our best knowledge, this is the first piece of evidence that implicates the involvement of SCs in the pathogenesis of adenomyosis induced by EMID.

Traditionally, the EMI has been viewed as important to the development of adenomyosis [11,58,59]. While uterine contractions have been well investigated in this regard [60,61], the nerve fibers have attracted scant, if any, attention, even though it has been proposed that injury to nerve fibers in the EMI due to difficult parturition and constipation is an antecedent of adenomyosis [22]. However, the plasticity of SCs plays an important role in the regeneration of peripheral nerves and tissue injury [28].

When EMID occurs following iatrogenic uterine procedures, the resulting tissue injury initiates tissue repair, featuring platelet aggregation and hemostasis, inflammation, hypoxia, proliferation and tissue remodeling [11]. Platelet aggregation can cause hypoxia, resulting in increased estrogen production in endometrial stromal cells [42]. Increased estrogen production and inflammatory cytokine secretion can provide a favorable microenvironment that is conducive to the differentiation of dedifferentiated SCs into endometrial epithelial cells (Wang et al., unpublished data). Once this happens, the SC-differentiated epithelial cells may undergo EMT under the combined action of hypoxia and elevated TGF-β and estrogen [17,62].

The ensuing tissue repair process also would result in the recruitment of macrophages into the wounding site (the EMI), resulting in the release of inflammatory cytokines and growth factors such as interleukin-1β (IL-1β), platelet-derived growth factor (PDGF), transforming growth factor β (TGF-β) and tumor necrosis factor (TNF-α) [63,64], all of which could promote the dedifferentiation of SCs [65,66,67,68]. Platelet activation and also proinflammatory cytokines such as TNF-α and IL-1β could induce cyclooxygenase-2 (COX-2), the gene encoding for the rate-limiting enzyme that produces prostaglandin E2 (PGE_2_), in endometrial stromal cells [69,70], resulting in increased production of estrogen [71]. Estrogen, coupled with PGE_2_, could also induce hyperperistalsis in the EMI, which could further exacerbate injury to the SCs as well as compromise the barrier function of EMI, making it more receptive to invading cells. Additionally, COX-2, in itself, also plays an important role in SCs demyelination [72].

One popular view regarding the pathogenesis of adenomyosis is the role of EMT, in which endometrial epithelial cells acquire their mobility and invasiveness, invade the myometrium through disrupted/breached EMI, establish the initial lesion and induce invagination [17,73,74,75,76]. Unfortunately, while the role of EMT in the pathogenesis is biologically plausible, so far, there have been no data that provide direct evidence in support for EMT.

To be fair, it is very challenging, if not ethically restrictive, to directly prove causal relationships in pathogenesis in humans. Since adenomyosis can and does occur in mouse spontaneously or by induction, mouse models of adenomyosis can be capitalized to aid our research into pathogenesis, especially when the EMID model is backed by ample epidemiological data [5]. In addition, it is also helpful not to be fixated on a particular culprit, as in any complex forensic investigations, but instead to have an open mind and cast a wider net whenever possible and practical [5].

Our findings strongly indicated that EMID-induced SC dedifferentiation contributed to the development of adenomyosis. However, the risk reduction, in terms of magnitude, is still moderate, especially for U0126. There are several explanations for this. First, the major goal of this study was to demonstrate that the perioperative suppression of SC dedifferentiation could reduce the risk of developing adenomyosis. As such, the intervention protocol that we used was by no means optimized in terms of dosage, timing and duration. While both the c-Jun and ERK pathways are rapidly activated after nerve injury [26,44], the elevated c-Jun protein levels last for 7–10 days, but the activation of ERK1/2 is reported to last for up to 1 month after injury [55,77]. Conceivably, the partial abrogation of the risk of adenomyosis by both compounds may be attributable to the partial suppression, entailed by the perioperative intervention, of the two pathways, at least in terms of shorter duration. In addition, the shorter duration of c-Jun activation relative to that of ERK may likely render more effective suppression by SP600125 than U0126, and a greater reduction in the adenomyosis risk, as seen in this study. This difference in duration that the two pathways manifest during SC dedifferentiation may account for the difference in the effects of the two compounds on the risk of developing adenomyosis.

Second, and alternatively, it is possible that SC dedifferentiation might not be the one and only determinant of the risk of developing adenomyosis resulting from EMID. In the wake of EMID induction, on top of SC dedifferentiation caused by nerve injury, resident endometrial epithelial cells may also be translocated into the myometrium by the tip of the procedure instrument or may simply mobilize and invade, through the EMT, into the myometrium through the breached barrier due to EMID, establishing the nascent adenomyotic focus. It is possible that dedifferentiated SCs and endometrial epithelial cells are both responsible for the seeding of the initial lesion. Still, given that injury-activated SCs can promote EMT and FMT through the paracrine action of TGF-β [78], it is very likely that SCs play a crucial and lasting role in the genesis of EMID-induced adenomyosis via promoting the EMT of both the SC-derived and endometrial epithelial cells that eventually invade into the myometrium. Therefore, further experiments are needed to ascertain the lineage of these epithelial-like cells within the myometrium, as well as the proportion of those of SC origin or endometrial origin.

The involvement of SCs in EMID-induced adenomyosis is supported by a recent study demonstrating that EMID induces the activation of signal transducer and activator of transcription 3 (STAT3) right after the injury, which persisted throughout the entire course of adenomyosis progression [13]. Indeed, STAT3 is activated after nerve injury [79], and it supports SC survival and is essential for the long-term maintenance of the phenotype of differentiated SCs [80]. That latter fact may suggest that, firstly, perioperative suppression of STAT3 could also be effective in reducing the risk of adenomyosis and, second, a complete elimination of the risk should require not only perioperative but also postoperative interventions.

Our findings have important scientific and clinical implications. Aside from the rounding up of a new culprit that is responsible for causing adenomyosis, which should be further interrogated to unravel the pathogenesis of adenomyosis, our study, in conjunction with our previous report [12], strongly indicates that perioperative intervention may be feasible in future iatrogenic uterine procedures. Given the ubiquity of these procedures nowadays, an effective intervention would substantially cut the risk of developing adenomyosis.

Our study has several strengths. First, we demonstrated the important role of SCs in the development of adenomyosis induced by EMID. When dedifferentiated, they could be trans-differentiated into endometrial epithelial cells and invade the myometrium, and, through further EMT, form a nascent adenomyotic focus. They could also facilitate the migration and invasion of endometrial epithelial cells, through inducing EMT, resulting in the invasion into the myometrium and establishment of the initial lesion. Second, our data are strongly indicative of the occurrence of EMT in epithelial cells (or, more precisely, granular epithelial cells) within the myometrium after EMID induction, providing not only direct but also more concrete evidence not only of the involvement of EMT but also, more importantly, of the involvement of SCs in the genesis of adenomyosis induced by EMID. Observations at different time points also provided a somewhat dynamic picture explaining just how perioperative intervention aimed at suppressing SC dedifferentiation could reduce the risk of adenomyosis caused by iatrogenic uterine procedures.

Of course, our study also has several notable limitations. First, we only used S100β and p75 as two markers for mature and dedifferentiated SCs, respectively. While S100β is exclusively expressed in nerve fibers [81] and p75 is a well-accepted marker for dedifferentiated SCs [55,56], p75 may also be expressed in the myometrium and endometrial stromal cells [81,82]. Using more than one marker would certainly provide further validation of our results. However, since the number of S100β+ and p75+ SCs within the EMI region were highly correlated (Figure 2C), which is consistent with our anticipated result that the increase in dedifferentiated SCs coincided with the decrease in mature SCs, the likelihood of massively misclassifying non-SCs as dedifferentiated ones is slim. Second, we did not investigate the impact of combined perioperative use of SP600125 and U0126, nor did we evaluate the effect of different dosages or the extended use of either compound on the incidence pf adenomyosis. In light of the role of SCs in the facilitation of EMT [30], FMT [78] and fibrogenesis [83], it is conceivable that continued suppression of SC dedifferentiation is likely to hinder the progression of adenomyosis through impeding EMT, FMT and fibrogenesis. Future studies are needed to clarify these issues. Third, since the ERK and JNK pathways are involved not only in SC dedifferentiation but also in other molecular processes such as inflammation [84,85] and EMT [86,87], future studies are warranted to delineate the role of SCs in EMID-induced adenomyosis more precisely, using, say, conditional knockout. Fourth, we made no attempt to ascertain the source of epithelial-like cells in the myometrium after EMID. In future studies, conditional knockout experiments could perhaps be conducted to trace the cellular lineage of the adenomyotic epithelial cells. Lastly, whether the density of nerve fibers, either PGP9.5+ or S100β+, was affected by perioperative administration of two inhibitors well beyond the perioperative window was not evaluated in our study. Therefore, future studies are needed to explore the effect of perioperative suppression of SC dedifferentiation on neuroprotection and neuro-regeneration, and their roles in the development of adenomyosis induced by EMID.

## 5. Conclusions

Our study has provided the first piece of evidence strongly implicating the involvement of SCs in the genesis of adenomyosis induced by EMID. In particular, it demonstrated that EMID-induced SC dedifferentiation is actively involved in the genesis of EMID-induced adenomyosis, and that perioperative suppression of SC dedifferentiation can reduce the risk of adenomyosis resulting from EMID in mice. Moreover, it highlighted the role of nerve fibers in the pathogenesis as well as the pathophysiology of adenomyosis, especially given the recent report on the involvement of neuropeptide and neurotransmitter receptors in adenomyosis [88]. More importantly, it underscores the prospect of effective perioperative intervention in reducing the risk of developing adenomyosis induced by iatrogenic uterine procedures. Given the importance of the pathogenesis of adenomyosis, our findings necessitate further independent validation. While the exact molecular mechanisms underlying EMID-induced adenomyosis and its possible intervention are still unclear, our study has nonetheless identified a culprit that is likely to be crucial in the pathogenesis of adenomyosis. Future investigations are badly needed to elucidate the molecular and cellular mechanisms of EMID-induced SC dedifferentiation and its role in the pathogenesis and pathophysiology of adenomyosis, and to devise effective strategies to reduce the risk of adenomyosis resulting from iatrogenic uterine procedures through perioperative interventions.

## Figures and Tables

**Table 1:** List of antibodies used in the IHC and immunofluorescence analyses.

Antibody Name	Catalog Number	Vendor Name and Location	Concentration
E-cadherin	3195S	CST, Boston, MA, USA	1:200
Vimentin	ARG66199	Arigobio, Shanghai, China	1:500
α-SMA	ab2675	Abcam, Cambridge, UK	1:100
S100β	90393	CST, Boston, MA, USA	1:200
p75	ab52987	Abcam, Cambridge, UK	1:100

**Table 2:** Incidence of adenomyosis and average grades of myometrial infiltration in mice.

Groups	Number and Sizes of Groups	# of Mice with Adenomyosis (Incidence of Adenomyosis) n (%)	Average Grades of Myometrial Infiltration		
			Average	Median	Range
CTL	13	13 (100%)	2.1 ± 0.7	2.2	0.8–3.0
SP600125	13	8 (61.5%)	0.6 ± 0.6	0.7	0.0–1.8
U0126	14	12 (85.7%)	1.1 ± 0.6	1.2	0.0–2.0

## Data Availability

The data presented in this study are available upon written request from the corresponding author explaining the use and purposes.

## References

[1] Upson K., Missmer S.A. (2020). Epidemiology of Adenomyosis. Semin. Reprod. Med..

[2] Benagiano G., Brosens I. (2006). History of adenomyosis. Best Pract. Res. Clin. Obstet. Gynaecol..

[3] Vannuccini S., Tosti C., Carmona F., Huang S.J., Chapron C., Guo S.W., Petraglia F. (2017). Pathogenesis of adenomyosis: An update on molecular mechanisms. Reprod. Biomed. Online.

[4] Garcia-Solares J., Donnez J., Donnez O., Dolmans M.M. (2018). Pathogenesis of uterine adenomyosis: Invagination or metaplasia?. Fertil. Steril..

[5] Wang X., Benagiano G., Liu X., Guo S.W. (2022). Unveiling the Pathogenesis of Adenomyosis through Animal Models. J. Clin. Med..

[6] Curtis K.M., Hillis S.D., Marchbanks P.A., Peterson H.B. (2002). Disruption of the endometrial-myometrial border during pregnancy as a risk factor for adenomyosis. Am. J. Obstet. Gynecol..

[7] Levgur M., Abadi M.A., Tucker A. (2000). Adenomyosis: Symptoms, histology, and pregnancy terminations. Obstet. Gynecol..

[8] Panganamamula U.R., Harmanli O.H., Isik-Akbay E.F., Grotegut C.A., Dandolu V., Gaughan J.P. (2004). Is prior uterine surgery a risk factor for adenomyosis?. Obstet. Gynecol..

[9] Parazzini F., Mais V., Cipriani S., Busacca M., Venturini P. (2009). Gise Determinants of adenomyosis in women who underwent hysterectomy for benign gynecological conditions: Results from a prospective multicentric study in Italy. Eur. J. Obstet. Gynecol. Reprod. Biol..

[10] Taran F.A., Weaver A.L., Coddington C.C., Stewart E.A. (2010). Understanding adenomyosis: A case control study. Fertil. Steril..

[11] Guo S.W. (2020). The Pathogenesis of Adenomyosis vis-a-vis Endometriosis. J. Clin. Med..

[12] Hao M., Liu X., Guo S.W. (2020). Adenomyosis in mice resulting from mechanically or thermally induced endometrial-myometrial interface disruption and its possible prevention. Reprod. Biomed. Online.

[13] Hiraoka T., Hirota Y., Aikawa S., Iida R., Ishizawa C., Kaku T., Hirata T., Fukui Y., Akaeda S., Matsuo M. (2022). Constant Activation of STAT3 Contributes to the Development of Adenomyosis in Females. Endocrinology.

[14] Hricak H., Alpers C., Crooks L.E., Sheldon P.E. (1983). Magnetic resonance imaging of the female pelvis: Initial experience. AJR Am. J. Roentgenol..

[15] Sofic A., Husic-Selimovic A., Carovac A., Jahic E., Smailbegovic V., Kupusovic J. (2016). The Significance of MRI Evaluation of the Uterine Junctional Zone in the Early Diagnosis of Adenomyosis. Acta Inform. Med..

[16] Leyendecker G., Wildt L. (2011). A new concept of endometriosis and adenomyosis: Tissue injury and repair (TIAR). Horm. Mol. Biol. Clin. Investig..

[17] Chen Y.J., Li H.Y., Huang C.H., Twu N.F., Yen M.S., Wang P.H., Chou T.Y., Liu Y.N., Chao K.C., Yang M.H. (2010). Oestrogen-induced epithelial-mesenchymal transition of endometrial epithelial cells contributes to the development of adenomyosis. J. Pathol..

[18] Shen M., Liu X., Zhang H., Guo S.W. (2016). Transforming growth factor beta1 signaling coincides with epithelial-mesenchymal transition and fibroblast-to-myofibroblast transdifferentiation in the development of adenomyosis in mice. Hum. Reprod..

[19] Cousins F.L., O D.F., Gargett C.E. (2018). Endometrial stem/progenitor cells and their role in the pathogenesis of endometriosis. Best Pract. Res. Clin. Obstet. Gynaecol..

[20] Tempest N., Jansen M., Baker A.M., Hill C.J., Hale M., Magee D., Treanor D., Wright N.A., Hapangama D.K. (2020). Histological 3D reconstruction and in vivo lineage tracing of the human endometrium. J. Pathol..

[21] Brosens I., Derwig I., Brosens J., Fusi L., Benagiano G., Pijnenborg R. (2010). The enigmatic uterine junctional zone: The missing link between reproductive disorders and major obstetrical disorders?. Hum. Reprod..

[22] Quinn M. (2007). Uterine innervation in fibroids: A qualitative study. J. Obstet. Gynaecol..

[23] Zhang X.M., Huang X., Xu H., Quinn M.J. (2014). Altered innervation of the fallopian tube in ectopic pregnancy. J. Obstet. Gynaecol..

[24] Krantz K.E. (1959). Innervation of the human uterus. Ann. N. Y. Acad. Sci..

[25] Barcena de Arellano M.L., Oldeweme J., Arnold J., Schneider A., Mechsner S. (2013). Remodeling of estrogen-dependent sympathetic nerve fibers seems to be disturbed in adenomyosis. Fertil. Steril..

[26] Jessen K.R., Mirsky R., Lloyd A.C. (2015). Schwann Cells: Development and Role in Nerve Repair. Cold Spring Harb. Perspect. Biol..

[27] Alvarez-Suarez P., Gawor M., Proszynski T.J. (2020). Perisynaptic schwann cells—The multitasking cells at the developing neuromuscular junctions. Semin. Cell Dev. Biol..

[28] Carr M.J., Johnston A.P. (2017). Schwann cells as drivers of tissue repair and regeneration. Curr. Opin. NeuroBiol..

[29] Stierli S., Imperatore V., Lloyd A.C. (2019). Schwann cell plasticity-roles in tissue homeostasis, regeneration, and disease. Glia.

[30] Sun L., Chen S., Chen M. (2022). Schwann Cells in the Tumor Microenvironment: Need More Attention. J. Oncol..

[31] Lee S.K., Wolfe S.W. (2000). Peripheral nerve injury and repair. J. Am. Acad. Orthop. Surg..

[32] Jessen K.R., Mirsky R. (2016). The repair Schwann cell and its function in regenerating nerves. J. Physiol..

[33] Joseph N.M., Mukouyama Y.S., Mosher J.T., Jaegle M., Crone S.A., Dormand E.L., Lee K.F., Meijer D., Anderson D.J., Morrison S.J. (2004). Neural crest stem cells undergo multilineage differentiation in developing peripheral nerves to generate endoneurial fibroblasts in addition to Schwann cells. Development.

[34] Kastriti M.E., Kameneva P., Kamenev D., Dyachuk V., Furlan A., Hampl M., Memic F., Marklund U., Lallemend F., Hadjab S. (2019). Schwann Cell Precursors Generate the Majority of Chromaffin Cells in Zuckerkandl Organ and Some Sympathetic Neurons in Paraganglia. Front. Mol. Neurosci..

[35] Adameyko I., Lallemend F., Aquino J.B., Pereira J.A., Topilko P., Muller T., Fritz N., Beljajeva A., Mochii M., Liste I. (2009). Schwann cell precursors from nerve innervation are a cellular origin of melanocytes in skin. Cell.

[36] Kaukua N., Shahidi M.K., Konstantinidou C., Dyachuk V., Kaucka M., Furlan A., An Z., Wang L., Hultman I., Ahrlund-Richter L. (2014). Glial origin of mesenchymal stem cells in a tooth model system. Nature.

[37] Dyachuk V., Furlan A., Shahidi M.K., Giovenco M., Kaukua N., Konstantinidou C., Pachnis V., Memic F., Marklund U., Muller T. (2014). Neurodevelopment. Parasympathetic neurons originate from nerve-associated peripheral glial progenitors. Science.

[38] Espinosa-Medina I., Outin E., Picard C.A., Chettouh Z., Dymecki S., Consalez G.G., Coppola E., Brunet J.F. (2014). Neurodevelopment. Parasympathetic ganglia derive from Schwann cell precursors. Science.

[39] Su Z., Niu W., Liu M.L., Zou Y., Zhang C.L. (2014). In vivo conversion of astrocytes to neurons in the injured adult spinal cord. Nat. Commun..

[40] Wang L.L., Su Z., Tai W., Zou Y., Xu X.M., Zhang C.L. (2016). The p53 Pathway Controls SOX2-Mediated Reprogramming in the Adult Mouse Spinal Cord. Cell Rep..

[41] Milichko V., Dyachuk V. (2020). Novel Glial Cell Functions: Extensive Potency, Stem Cell-Like Properties, and Participation in Regeneration and Transdifferentiation. Front. Cell Dev. Biol..

[42] Qi Q., Liu X., Zhang Q., Guo S.W. (2020). Platelets induce increased estrogen production through NF-kappaB and TGF-beta1 signaling pathways in endometriotic stromal cells. Sci. Rep..

[43] Shaw T.J., Martin P. (2009). Wound repair at a glance. J. Cell Sci..

[44] Nocera G., Jacob C. (2020). Mechanisms of Schwann cell plasticity involved in peripheral nerve repair after injury. Cell. Mol. Life Sci..

[45] Harrisingh M.C., Perez-Nadales E., Parkinson D.B., Malcolm D.S., Mudge A.W., Lloyd A.C. (2004). The Ras/Raf/ERK signalling pathway drives Schwann cell dedifferentiation. EMBO J..

[46] Blom C.L., Martensson L.B., Dahlin L.B. (2014). Nerve injury-induced c-Jun activation in Schwann cells is JNK independent. Biomed. Res. Int..

[47] Council N.R. (2011). Guide for the Care and Use of Laboratory Animals.

[48] Zhang H., Niu X., Qian Z., Qian J., Xuan B. (2015). The c-Jun N-terminal kinase inhibitor SP600125 inhibits human cytomegalovirus replication. J. Med. Virol..

[49] Zhou Q., Wang M., Du Y., Zhang W., Bai M., Zhang Z., Li Z., Miao J. (2015). Inhibition of c-Jun N-terminal kinase activation reverses Alzheimer disease phenotypes in APPswe/PS1dE9 mice. Ann. Neurol..

[50] Ouyang H., Yang H.S., Yu T., Shan T.D., Li J.Y., Huang C.Z., Zhong W., Xia Z.S., Chen Q.K. (2016). MEK/ERK pathway activation by insulin receptor isoform alteration is associated with the abnormal proliferation and differentiation of intestinal epithelial cells in diabetic mice. Mol. Cell. Biochem..

[51] Marampon F., Bossi G., Ciccarelli C., Di Rocco A., Sacchi A., Pestell R.G., Zani B.M. (2009). MEK/ERK inhibitor U0126 affects in vitro and in vivo growth of embryonal rhabdomyosarcoma. Mol. Cancer Ther..

[52] Bird C.C., McElin T.W., Manalo-Estrella P. (1972). The elusive adenomyosis of the uterus--revisited. Am. J. Obstet. Gynecol..

[53] Raponi E., Agenes F., Delphin C., Assard N., Baudier J., Legraverend C., Deloulme J.C. (2007). S100B expression defines a state in which GFAP-expressing cells lose their neural stem cell potential and acquire a more mature developmental stage. Glia.

[54] Fregoso S.P., Hoover D.B. (2012). Development of cardiac parasympathetic neurons, glial cells, and regional cholinergic innervation of the mouse heart. Neuroscience.

[55] Jessen K.R., Arthur-Farraj P. (2019). Repair Schwann cell update: Adaptive reprogramming, EMT, and stemness in regenerating nerves. Glia.

[56] Napoli I., Noon L.A., Ribeiro S., Kerai A.P., Parrinello S., Rosenberg L.H., Collins M.J., Harrisingh M.C., White I.J., Woodhoo A. (2012). A central role for the ERK-signaling pathway in controlling Schwann cell plasticity and peripheral nerve regeneration in vivo. Neuron.

[57] Choi S.J., Park S.Y., Shin Y.H., Heo S.H., Kim K.H., Lee H.I., Kim J.K. (2021). Mesenchymal Stem Cells Derived from Wharton’s Jelly Can Differentiate into Schwann Cell-Like Cells and Promote Peripheral Nerve Regeneration in Acellular Nerve Grafts. Tissue Eng. Regen. Med..

[58] Zhai J., Vannuccini S., Petraglia F., Giudice L.C. (2020). Adenomyosis: Mechanisms and Pathogenesis. Semin. Reprod. Med..

[59] Zhang Y., Zhou L., Li T.C., Duan H., Yu P., Wang H.Y. (2014). Ultrastructural features of endometrial-myometrial interface and its alteration in adenomyosis. Int. J. Clin. Exp. Pathol..

[60] Huang M., Li X., Guo P., Yu Z., Xu Y., Wei Z. (2017). The abnormal expression of oxytocin receptors in the uterine junctional zone in women with endometriosis. Reprod. Biol. Endocrinol..

[61] Maruyama S., Imanaka S., Nagayasu M., Kimura M., Kobayashi H. (2020). Relationship between adenomyosis and endometriosis; Different phenotypes of a single disease?. Eur. J. Obstet. Gynecol. Reprod. Biol..

[62] Lin Y.T., Wu K.J. (2020). Epigenetic regulation of epithelial-mesenchymal transition: Focusing on hypoxia and TGF-beta signaling. J. Biomed. Sci..

[63] Shapouri-Moghaddam A., Mohammadian S., Vazini H., Taghadosi M., Esmaeili S.A., Mardani F., Seifi B., Mohammadi A., Afshari J.T., Sahebkar A. (2018). Macrophage plasticity, polarization, and function in health and disease. J. Cell. Physiol..

[64] Murdoch C., Muthana M., Lewis C.E. (2005). Hypoxia regulates macrophage functions in inflammation. J. Immunol..

[65] Basu S., Choudhury I.N., Nazareth L., Chacko A., Shelper T., Vial M.L., Ekberg J.A.K., St John J.A. (2022). In vitro modulation of Schwann cell behavior by VEGF and PDGF in an inflammatory environment. Sci. Rep..

[66] Balakrishnan A., Belfiore L., Chu T.H., Fleming T., Midha R., Biernaskie J., Schuurmans C. (2020). Insights Into the Role and Potential of Schwann Cells for Peripheral Nerve Repair From Studies of Development and Injury. Front. Mol. Neurosci..

[67] Chen G., Luo X., Wang W., Wang Y., Zhu F., Wang W. (2019). Interleukin-1beta Promotes Schwann Cells De-Differentiation in Wallerian Degeneration via the c-JUN/AP-1 Pathway. Front. Cell. Neurosci..

[68] Chu L.W., Cheng K.I., Chen J.Y., Cheng Y.C., Chang Y.C., Yeh J.L., Hsu J.H., Dai Z.K., Wu B.N. (2020). Loganin prevents chronic constriction injury-provoked neuropathic pain by reducing TNF-alpha/IL-1beta-mediated NF-kappaB activation and Schwann cell demyelination. Phytomedicine.

[69] Wu Y., Guo S.W. (2007). Suppression of IL-1beta-induced COX-2 expression by trichostatin A (TSA) in human endometrial stromal cells. Eur. J. Obstet. Gynecol. Reprod. Biol..

[70] Ding D., Liu X., Duan J., Guo S.W. (2015). Platelets are an unindicted culprit in the development of endometriosis: Clinical and experimental evidence. Hum. Reprod..

[71] Bulun S.E., Lin Z., Imir G., Amin S., Demura M., Yilmaz B., Martin R., Utsunomiya H., Thung S., Gurates B. (2005). Regulation of aromatase expression in estrogen-responsive breast and uterine disease: From bench to treatment. Pharmacol. Rev..

[72] Cao P., Zhang H., Meng H., Cheng Y., Xu H., Zang S., Li Z., Cui J., Li Y. (2020). Celecoxib Exerts a Therapeutic Effect Against Demyelination by Improving the Immune and Inflammatory Microenvironments. J. Inflamm. Res..

[73] Oh S.J., Shin J.H., Kim T.H., Lee H.S., Yoo J.Y., Ahn J.Y., Broaddus R.R., Taketo M.M., Lydon J.P., Leach R.E. (2013). beta-Catenin activation contributes to the pathogenesis of adenomyosis through epithelial-mesenchymal transition. J. Pathol..

[74] Khan K.N., Kitajima M., Hiraki K., Fujishita A., Nakashima M., Masuzaki H. (2015). Involvement of hepatocyte growth factor-induced epithelial-mesenchymal transition in human adenomyosis. Biol. Reprod..

[75] Qi S., Zhao X., Li M., Zhang X., Lu Z., Yang C., Zhang C., Zhang H., Zhang N. (2015). Aberrant expression of Notch1/numb/snail signaling, an epithelial mesenchymal transition related pathway, in adenomyosis. Reprod. Biol. Endocrinol..

[76] Yoo J.Y., Ku B.J., Kim T.H., Il Ahn J., Ahn J.Y., Yang W.S., Lim J.M., Taketo M.M., Shin J.H., Jeong J.W. (2020). beta-catenin activates TGF-beta-induced epithelial-mesenchymal transition in adenomyosis. Exp. Mol. Med..

[77] Agthong S., Kaewsema A., Tanomsridejchai N., Chentanez V. (2006). Activation of MAPK ERK in peripheral nerve after injury. BMC Neurosci..

[78] Parfejevs V., Debbache J., Shakhova O., Schaefer S.M., Glausch M., Wegner M., Suter U., Riekstina U., Werner S., Sommer L. (2018). Injury-activated glial cells promote wound healing of the adult skin in mice. Nat. Commun..

[79] Wang H., Zhu H., Guo Q., Qian T., Zhang P., Li S., Xue C., Gu X. (2017). Overlapping Mechanisms of Peripheral Nerve Regeneration and Angiogenesis Following Sciatic Nerve Transection. Front. Cell. Neurosci..

[80] Benito C., Davis C.M., Gomez-Sanchez J.A., Turmaine M., Meijer D., Poli V., Mirsky R., Jessen K.R. (2017). STAT3 Controls the Long-Term Survival and Phenotype of Repair Schwann Cells during Nerve Regeneration. J. Neurosci..

[81] Chiang S. (2021). S100 and Pan-Trk Staining to Report NTRK Fusion-Positive Uterine Sarcoma: Proceedings of the ISGyP Companion Society Session at the 2020 USCAP Annual Meeting. Int. J. Gynecol. Pathol..

[82] Green A.R., Edwards R.E., Greaves P., White I.N. (2003). Comparison of the effect of oestradiol, tamoxifen and raloxifene on nerve growth factor-alpha expression in specific neonatal mouse uterine cell types using laser capture microdissection. J. Mol. Endocrinol..

[83] Direder M., Weiss T., Copic D., Vorstandlechner V., Laggner M., Pfisterer K., Mildner C.S., Klas K., Bormann D., Haslik W. (2022). Schwann cells contribute to keloid formation. Matrix Biol..

[84] Wang Y., He G., Tang H., Shi Y., Kang X., Lyu J., Zhu M., Zhou M., Yang M., Mu M. (2019). Aspirin inhibits inflammation and scar formation in the injury tendon healing through regulating JNK/STAT-3 signalling pathway. Cell Prolif..

[85] Dong Q., Jie Y., Ma J., Li C., Xin T., Yang D. (2019). Renal tubular cell death and inflammation response are regulated by the MAPK-ERK-CREB signaling pathway under hypoxia-reoxygenation injury. J. Recept. Signal Transduct. Res..

[86] Geng Q., Li Z., Li X., Wu Y., Chen N. (2021). LncRNA NORAD, sponging miR-363-3p, promotes invasion and EMT by upregulating PEAK1 and activating the ERK signaling pathway in NSCLC cells. J. Bioenerg. Biomembr..

[87] Sheng W., Shi X., Lin Y., Tang J., Jia C., Cao R., Sun J., Wang G., Zhou L., Dong M. (2020). Musashi2 promotes EGF-induced EMT in pancreatic cancer via ZEB1-ERK/MAPK signaling. J. Exp. Clin. Cancer Res..

[88] Xu X., Cai X., Liu X., Guo S.W. (2021). Possible involvement of neuropeptide and neurotransmitter receptors in Adenomyosis. Reprod. Biol. Endocrinol..

